# BEHAVIORAL MARKERS OF RESILIENCE IN CARE PARTNERS OF PERSONS WITH DEMENTIA: A THEMATIC ANALYSIS FROM A SCOPING REVIEW

**DOI:** 10.1093/geroni/igz038.2211

**Published:** 2019-11-08

**Authors:** Yuanjin Zhou, Avery O’Hara, Emily Ishado, Soo Borson, Tatiana Sadak

**Affiliations:** 1 University of Washington School of Social Work, Seattle, Washington, United States; 2 University of Washington School of Nursing, Seattle, Washington, United States; 3 University of Washington School of Medicine, Seattle, Washington, United States

## Abstract

Caring for a person with dementia (PWD) requires commitment, flexibility, and resilience - the ability to endure and recover from stressors that arise during the caring process. However, it is unknown what behaviors can indicate resilience in care partners (CPs) of PWDs. We examined 46 peer-reviewed articles (1990 to 2018) that included measures or definitions of resilience in CPs of PWDs. Our goal is to identify resilience-related behaviors and create a behavior-based model/framework for CPs of PWDs. Three major themes emerged: (1) Problem-response behaviors (problem-identifying, problem-solving, problem-distancing, learning, and reflection); (2) self-growth behaviors (self-care, creative/spiritual activities, and developing/maintaining meaningful social relationships); (3) help-related behaviors (help-seeking and help-receiving). These findings informed the development of a behavior-based Care Partner Resilience (CPR) measure. Future steps in this research include evaluating to what extent behaviors in the CPR framework are associated with CPs’ self-assessed resilience and can predict CPs’ resilience following specific caregiving-related stressors.

